# The MOTION randomized controlled trial for treatment of lumbar spinal stenosis using the percutaneous mild® procedure: 5-year results

**DOI:** 10.1016/j.inpm.2026.100768

**Published:** 2026-05-12

**Authors:** Timothy R. Deer, Mayank Gupta, Sherif Costandi, Brent Chafin, Sayed Wahezi, Samyadev Datta, Dawood Sayed

**Affiliations:** a The Spine & Nerve Centers of the Virginias, 400 Court Street, Suite 100, Charleston, WV, 25301, USA; b Kansas Pain Management / Neuroscience Research Center, LLC, 10995 Quivira Rd, Overland Park, KS, 66210, USA; c Cleveland Clinic, 9500 Euclid Ave., Cleveland, OH, 44195, USA; d Vidant Roanoke-Chowan Hospital, 500 S. Academy St, Ahoskie, NC, 27910, USA; e Montefiore Medical Center, 111 East 210th Street, Bronx, NY, 10461, USA; f Holy Name Medical Center, 718 Teaneck Road, Teaneck, NJ, 07666, USA; g University of Kansas Pain Clinic, 3901 Rainbow Blvd, Kansas City, KS, 66160, USA

**Keywords:** Mild, Hypertrophic Ligamentum Flavum (HLF), Neurogenic claudication, Lumbar Spinal Stenosis (LSS), Low back pain, Percutaneous Image-Guided Lumbar Decompression (PILD)

## Abstract

**Objective:**

Long-term outcomes of percutaneous image-guided lumbar decompression for treatment of lumbar spinal stenosis with neurogenic claudication secondary to hypertrophic ligamentum flavum were assessed using extended follow-up of the treatment group in the MOTION prospective, multicenter randomized controlled trial. Originally performed with a control group consisting solely of conventional medical management (CMM-Alone), follow-up of the treatment group was extended to five years and analyzed as a modified intent-to-treat group.

**Methods:**

Spinal decompression was performed using the mild® Procedure (Stryker Corporation, Portage, MI, USA), with the treatment group also receiving CMM with no restrictions post-procedure (mild + CMM). Subjective outcomes were measured using validated patient-reported questionnaires including the Oswestry Disability Index (ODI), Zurich Claudication Questionnaire (ZCQ), and Numeric Pain Rating Scale (NPRS). Objective measurements included a validated Walking Tolerance Test (WTT), the rate of subsequent lumbar spine interventions (SLSI), and the occurrence of adverse events. An ad hoc analysis comparing 5-year outcomes for patients who were at least 65 years of age at the time of treatment to those of younger patients was also performed. In addition, long-term (4-year) outcomes were assessed for CMM-Alone patients who subsequently received the mild Procedure for relief of ongoing symptoms (crossover group).

**Results:**

As with the 1-, 2- and 3-year follow-up visits, all outcomes at the 5-year visit for the mild + CMM group remained significantly improved over baseline (N = 34, p < 0.0001), with ODI, NPRS back and leg, and ZCQ Symptom Severity and Physical Function improving by 20.6, 2.5, 4.6, 0.9, and 0.7, respectively. Walking times increased 326% from baseline, with three additional SLSI performed since the 3-year follow-up. No device- or procedure-related adverse events were reported over the entire 5-year follow-up period. At 5 years post-treatment, no significant differences in outcomes between older and younger patients were seen for any outcome. No significant differences were found in the crossover group when compared to the mild + CMM group using 4-year follow-up results.

**Conclusions:**

The use of the percutaneous mild Procedure, together with CMM, is shown to provide a safe, effective and durable treatment for symptomatic LSS. The procedure appears effective regardless of patient age at the time of treatment. Further, the ability of the mild Procedure to improve patient outcomes does not appear to be affected in those patients whose treatment is delayed by continued use of CMM.

## 1 Introduction

Nearly half of the 103 million cases of lumbar spinal stenosis (LSS) diagnosed globally each year result from the narrowing of the spinal canal due to hypertrophied ligamentum flavum (HLF) [1,2], with the incidence of LSS resulting from HLF significantly correlated with age [1,3]. The narrowed spinal canal can compress neural elements and result in back, buttock or leg pain usually accompanied by neurogenic claudication (NC). Initial treatments for LSS are conservative and can include oral pain medications, physical therapy and exercise programs [4].

Percutaneous image-guided lumbar decompression (PILD) was developed as a minimally invasive alternative to surgical decompression for treatment of LSS with HLF and has been recommended as a first-level intervention for patients who are non-responsive to conservative therapies [[4], [5], [6], [7]]. The PILD approach using the mild® Procedure and instruments (Stryker Corporation, Portage, MI) has been studied in numerous retrospective and prospective clinical investigations since 2008 [[8], [9], [10]]. These and other studies have shown the mild Procedure to be an effective surgical technique for treating LSS with HLF with a lower rate of adverse events compared to other surgical decompression techniques including open surgical decompression, interspinous spacers and intervertebral fusion [8,[10], [11], [12]].

The MOTION study is a prospective, randomized trial comparing the mild procedure in combination with conventional medical management (mild + CMM) to the use of CMM only (CMM-Alone) for the treatment of LSS with NC resulting from HLF. The study design recognizes that clinicians are likely to recommend CMM as needed for patients subsequent to minimally invasive surgical intervention. The MOTION study protocol allows for CMM-Alone patients to cross over to receive the mild procedure after their one-year follow-up visit, and for all patients in the mild + CMM and cross-over groups to be evaluated annually through an extended 5-year follow-up. The study results of the 4- and 5-year follow-up visits are presented in this report. The results for the follow-up intervals of 6 months, 1-, 2- and 3-years have been previously reported [[13], [14], [15], [16]].

## 2 Methods

Study enrollment was limited to patients aged 50 – 80 years with LSS as assessed independently via magnetic resonance or computed tomography images and having NC symptoms for a minimum of 3 months. The complete list of inclusion/exclusion criteria has been previously reported [17]. Patients were randomly assigned to either the treatment group (mild + CMM) or the control group (CMM-Alone) in a 1:1 ratio. While the study was originally designed for patient participation through a 2-year follow-up, an additional three years of data collection for the mild + CMM group was added via a protocol amendment to evaluate long-term outcomes. Each patient provided informed consent to participate in the MOTION study, including for the 3- to 5-year extended follow-up. After 12 months of study participation, patients in the CMM-Alone group were allowed to seek mild treatment as part of a cross-over group.

The study, registered as NCT03610737 with the Clinical Trial Registry (clinicaltrials.gov), was performed following Institutional Review Board approval and in compliance with the International Conference on Harmonization guidelines for Good Clinical Practices.

### 2.1 Conventional medical management

Study investigators provided each participant with an individualized CMM treatment plan. These plans included therapies such as analgesics, exercise, physical therapy and epidural steroid injections, and were used as the sole treatments for the CMM-Alone group or in addition to the mild Procedure in the mild + CMM group.

### 2.2 The mild procedure

Percutaneous image-guided lumbar decompression using the mild Procedure is performed from a posterior approach to the spine using local anesthetic and light sedation. A single 5.1 mm incision is made for initial fluoroscopic placement of a stabilizing portal. Through continued use of fluoroscopic imaging, a specialized rongeur is passed through the portal to remove lamina sufficient to access the ligamentum flavum. The hypertrophied ligamentum flavum is subsequently debulked using additional specialized rongeurs, serving to decompress the adjacent neural tissues. The procedure has been previously described in detail [17].

### 2.3 Outcome measures

Patient outcomes were measured using both subjective patient-reported outcomes and an objective performance measure. The primary outcome was pain and functional disability as measured with the Oswestry Disability Index (ODI). Secondary outcomes included both back and leg pain as measured via the Numeric Pain Rating Scale (NPRS) and symptom severity and physical function related to LSS using the Zurich Claudication Questionnaire (ZCQ). A validated Walking Tolerance Test (WTT) to assess performance was also used as a secondary outcome. In this test, the time for patients to experience severe symptoms of NC while walking at their own pace was recorded, to a maximum of 15 min.

All device-related and procedure-related adverse events (AEs) and serious adverse events (SAEs) were recorded and adjudicated by an independent clinical event monitor. Any subsequent lumbar spine interventions (SLSI) for study patients with persistent symptoms were also recorded.

### 2.4 Analysis methods

Results of continuous data are presented using means and standard deviations. Comparisons of mean values were performed using a two-tailed *t*-test with a significance level set at 0.05. Between-group analyses assumed unequal sample variances. For within-group analyses, paired t-tests were used with dependent samples. Frequency counts and percentages are used to summarize categorical data using Fisher's exact test. Summary calculations for AE/SAE events included not only the number of patients experiencing the event but also the total number of events they encountered to accommodate patients who experienced multiple events. Data from the 3- to 5-year extended follow-up period was analyzed as a modified intent-to-treat (mITT) population. All analyses were performed using VassarStats (http://www.vassarstats.net)/).

## 3 Results

### 3.1 Patient characteristics

**Fig. 1:**
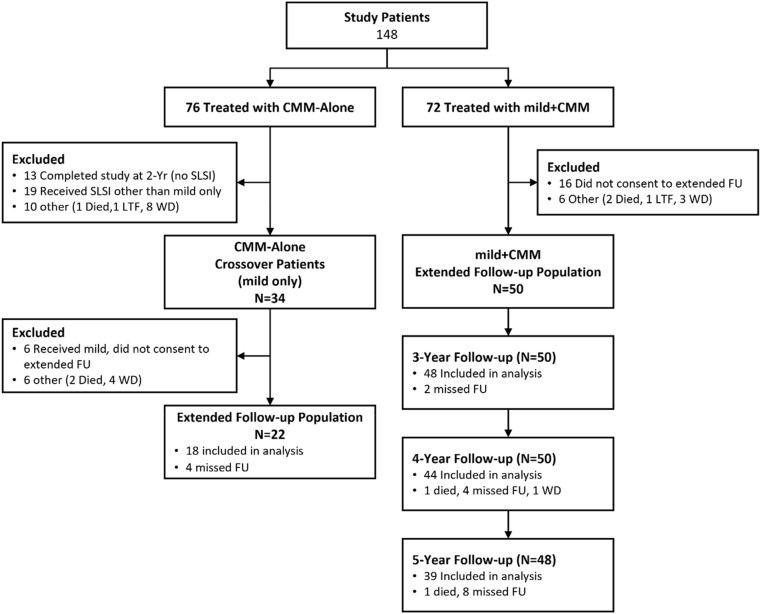
Extended follow-up study patient flow. SLSI = subsequent lumbar spine intervention, FU = follow-up, LTF = Lost to follow-up, WD=Withdrew.

The MOTION study included a total of 148 patients who met the study selection criteria and received treatment from September 2018 through December 2019; 72 patients in the mild + CMM group and 76 patients in the CMM-Alone group. Of the 50 patients in the mild-CMM group who consented to the extended follow-up, 48, 44 and 39 patients were evaluated at the 3-, 4- and 5-year follow-up visits, respectively, with the remaining patients not included in the analysis due to missed visits, withdrawal from the study and death (Fig. 1). The mean age for patients in the mild + CMM and CMM-Alone groups was 64.7 years and 66.8 years, respectively. Previous reports have described the complete patient demographics as well as the baseline values for all measured parameters. There were no significant differences between the treatment and control groups for these measures at baseline [14]. Table 1 lists the CMM treatments utilized by the patients in this study.

**Table 1:** CMM treatments utilized by MOTION patients.

Activity restriction	Hip/knee injections	Sacroiliac joint injection
Acupuncture	Home exercise	Senior stability class
Aquatic therapy	Massage Therapy	Stem cell therapy
Back brace	Inversion table	Stretching
Bed rest	Medial branch injection	Swimming
Biking	Occupational therapy	Trigger point injection
Chiropractic adjustment	Pain medication	TENS unit
Epidural steroid injection	Physical therapy	Weight management plan
Facet joint injection	PRP therapy	Wellness center
GTB injection	Radiofrequency ablation	Walking aid
Heat and/or Ice therapy	Rhizotomy	Yoga

Abbreviations: GTB, greater trochanteric bursa, PRP, platelet-rich plasma, TENS, Transcutaneous Electrical Nerve Stimulation.

Of the 34 CMM-Alone patients who subsequently elected to receive the mild Procedure without any other surgical intervention for treatment of their unresolved symptoms, four subsequently received other surgical interventions for recurrence of low back pain. Eighteen of the remaining 22 patients in the crossover group who agreed to participate in extended follow-up completed both the 2-year visit (22 – 28 months) and the 4-year visit (>42 months), with 15 of these patients also seen at 36 months (32 – 40 months). The mean time to crossover for these 18 patients was 14.8 months (range: 1.6-28.9 months) before receiving the mild Procedure.

### 3.2 Outcome measures

**Fig. 2:**
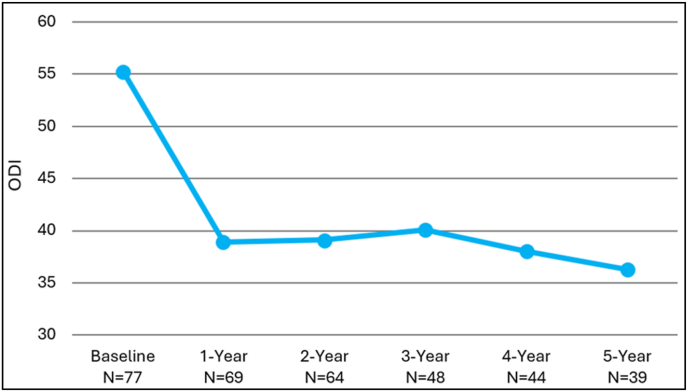
Oswestry Disability Index (ODI) mean score for the mild + CMM group.

The mean ODI scores for patients at their 4- and 5-year follow-up visits showed a statistically significant improvement over baseline scores by 19.4 and 20.6 points, respectively (p < 0.0001) (Fig. 2).

**Fig. 3:**
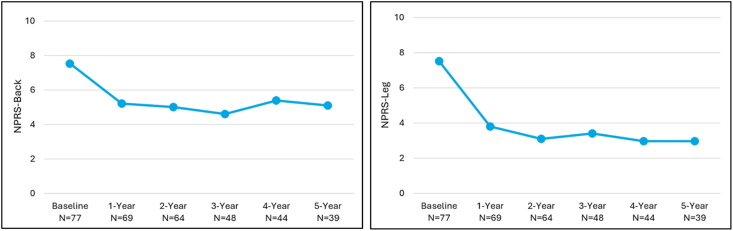
Numeric Pain Rating Scale (NPRS) mean scores for back and leg pain for the mild + CMM group.

Mean back and leg pain NPRS scores were also significantly improved over baseline, with back score improvements of 2.3 points and 2.5 points and leg score improvements of 4.7 and 4.6 points at 4- and 5-years, respectively (p < 0.0001) (Fig. 3).

**Fig. 4:**
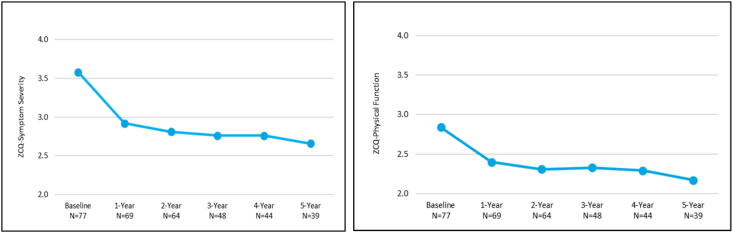
Zurich Claudication Questionnaire (ZCQ) mean scores for symptom severity and physical function for the mild + CMM group.

The ZCQ scores reflected those of the ODI and NPRS scores. Mean symptom severity scores of 0.8 and 0.9 points and mean physical function scores of 0.6 and 0.7 points at the 4- and 5-year follow-up visits were all statistically significant when compared to baseline scores (p < 0.0001) (Fig. 4).

**Fig. 5:**
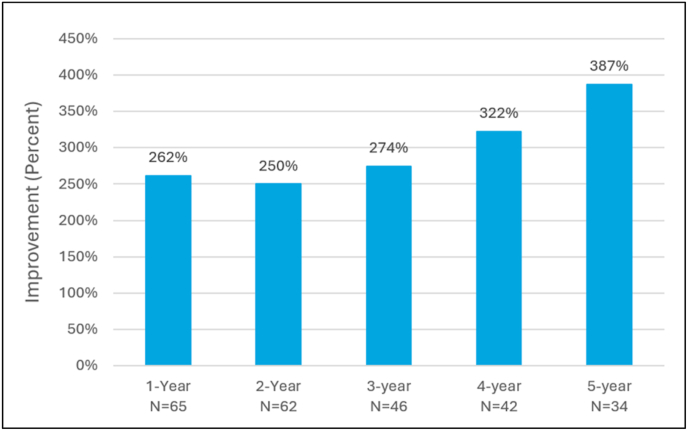
15-Minute Walking Tolerance Test mean percent improvement compared to baseline for the mild + CMM group.

Patients also experienced significant improvements in the mean length of time that they were able to walk in the objective Walking Tolerance Test. The mean improvement in walking times in this test was measured at 322% and 387% of the baseline walking times at the 4- and 5-year follow-up visits, respectively (p < 0.0001) (Fig. 5).

No device- or procedure-related adverse events were reported through 5 years for the mild + CMM group. As previously reported by Constandi et al. [16], 10 patients in the mild + CMM group had undergone an SLSI by the 3-year follow-up visit (with 5 of those occurring near the time of the initial mild treatment). Three additional patients received an SLSI by the 5-year follow-up. The 13 SLSI treatments included implantation of an interspinous spacer (2), use of a neurostimulator (2), fusion (2), an additional mild procedure (2), lumbar decompression (1), hemilaminotomy (1), laminectomy (1) and adhesiolysis (1). One patient had treatment of interspinous spacer and laminectomy.

Two patients experienced unrelated serious adverse events, specifically gastrointestinal bleeding, during the 3- and 4-year extended follow-up period. One of these patients was later found to have died from an unknown cause sometime between the 4- and 5-year follow-up visits.

### 3.3 Subset analysis – Patient age

A subset analysis of the mild + CMM patients who were at least 65 years old at the time of treatment and completed 5 years of follow-up found mean improvements in ODI, NPRS (leg), NPRS (back), ZCQ Symptom Severity and ZCQ Physical Function scores of 14.9, 5.3, 2.3, 0.8 and 0.7 points, respectively, in 18 patients. The mean improvement in WTT was 238% for the 16 patients in this group who completed the test. Each of these improvements over baseline were statistically significant. Table 2 compares these outcomes to mild + CMM patients who were less than 65 years old at time of treatment, for whom each improvement over baseline was also statistically significant. While the improvements experienced by the younger age group were generally greater than those seen in the older group, none of the differences between the two groups were significant.

**Table 2:** Outcome Improvement Comparison for mild + CMM Group at 1-5 Year Follow-ups.

Outcome Improvement (Mean ± SD) P-value (within group)	1-Year	2-Year	3-Year	4-Year	5-Year
Sample Size:	N = 69	N = 64	N = 48	N = 44	N = 39
ODI	16.5 ± 19.3	17.0 ± 21.4	16.9 ± 20.5	19.4 ± 20.5	20.6 ± 17.8
p-value	<0.0001	<0.0001	<0.0001	<0.0001	<0.0001
NPRS					
Back	2.4 ± 2.8	2.6 ± 3.0	3.0 ± 2.8	2.3 ± 2.9	2.5 ± 2.8
p-value	<0.0001	<0.0001	<0.0001	<0.0001	<0.0001
Leg	3.8 ± 3.1	4.5 ± 3.0	4.3 ± 2.9	4.7 ± 2.9	4.6 ± 2.9
p-value	<0.0001	<0.0001	<0.0001	<0.0001	<0.0001
ZCQ					
Symptom Severity	0.7 ± 0.8	0.8 ± 1.0	0.8 ± 1.0	0.8 ± 0.9	0.9 ± 1.0
p-value	<0.0001	<0.0001	<0.0001	<0.0001	<0.0001
Physical Function	0.5 ± 0.7	0.6 ± 0.8	0.6 ± 0.7	0.6 ± 0.7	0.7 ± 0.7
p-value	<0.0001	<0.0001	0.0001	<0.0001	<0.0001
**Sample Size:**	**N=65**^a^	**N=62**^a^	**N=46**^a^	**N=42**^a^	**N=34**^a^
Walking Tolerance Test	262%	250%	274%	322%	326%
p-value	<0.0001	<0.0001	0.0021	<0.0001	<0.0001

a Not all patients who completed 5-year follow-up questionnaires performed the WTT.

Of the thirty eight patients in the mild + CMM group who were at least 65 years old at the time of treatment, nine (23.7%) received some form of SLSI over the 5 years of follow-up. While only four (11.8%) of the thirty four patients in the group who were younger than 65 years old experienced an SLSI for the same time period, the difference in SLSI rates between the two age groups is not significant (p = 0.2308). Three patients in the older group underwent a decompression and/or fusion surgical treatment as their SLSI (7.9%) compared to two patients in the younger group (5.9%), with the difference being not significant (p = 1.000).

### 3.4 Subset analysis – crossover group

An additional subset analysis focusing on the crossover patients was also performed. Table 3 presents the mean improvements seen at each of the crossover group follow-up visits over baseline values. The degree of improvement was generally consistent with that seen in the mild + CMM group at similar follow-up time points, and as with the mild + CMM group, improvements for all measures over baseline values at all follow-ups were significant.

**Table 3:** 5-Year Outcome Comparison Between mild + CMM Patients Under 65 and 65 or Older.

Outcome Improvement (Mean ± SD) P-value (within group)	<65 Years	≥65 Years	P-Value Between Groups
Sample Size:	N = 21	N = 18	
ODI	25.4 ± 15.2	14.9 ± 19.4	0.0652
p-value	<0.0001	0.0045	
NPRS			
Back	2.6 ± 2.8	2.3 ± 2.9	0.7135
p-value	0.0002	0.0041	
Leg	4.0 ± 2.6	5.3 ± 3.2	0.1759
p-value	<0.0001	<0.0001	
ZCQ			
Symptom Severity	1.0 ± 0.8	0.8 ± 1.1	0.6553
p-value	<0.0001	0.0064	
Physical Function	0.8 ± 0.6	0.7 ± 0.7	0.5654
p-value	<0.0001	0.0013	
**Sample Size:**	**N=18**^a^	**N=16**^a^	
Walking Tolerance Test	520%	238%	0.0933
p-value	0.0004	0.0083	

a Not all patients who completed 5-year follow-up questionnaires performed the WTT.

## 4 Discussion

Patients in the mild + CMM group experienced significant levels of improvement over baseline for ODI, NPRS, ZCQ and WTT when evaluated not only at six months after the mild treatment but also at their 1- and 2-year follow-up visits [14,15]. Patients in this group maintained these significant levels of improvement for 3 additional years of extended follow-up, with mean improvements in ODI, NPRS (leg), NPRS (back), scores of 20.6, 4.6 and 2.5 at the 5-year follow-up, respectively. The mean improvement for ZCQ Symptom Severity, ZCQ Physical Function and WTT scores at the 5-year follow-up were 0.9, 0.7 and 326%, respectively.

Patients who received a SLSI (n = 9) during the 5-year follow-up were included in the modified intent-to-treat analysis. To determine the influence of including these nine patients in the analysis, the results from the mITT (n = 39) were compared to those who did not receive an SLSI (n = 30). The magnitude of improvement in the no-SLSI subgroup was similar to that observed overall for ODI (20.5 ± 17.9; p = 0.9762), NPRS back (2.5 ± 2.8; p = 0.9523), NPRS leg (4.3 ± 2.9; p = 0.7200), ZCQ symptom severity (1.0 ± 1.0; p = 0.7880), and ZCQ physical function (0.7 ± 0.7; p = 0.8577). Walking Tolerance Test improvements were also similar (399%; p = 0.9207), noting that not all patients who completed 5-year follow-up questionnaires performed the WTT (n = 29 for the no-SLSI subgroup). Overall, no statistically significant differences were observed between analyses with and without SLSI, indicating that inclusion of patients with SLSI did not amplify the long-term impact of the mild procedure on patient outcome.

These outcomes are similar to those resulting from open surgical decompression without fusion in patients with similar study inclusion criteria at 5 years post-treatment. Hermansen et al. found overall mean improvements in ODI, NPRS (leg) and NPRS (back) scores of 18.6, 3.4 and 2.6 points, respectively, in 318 – 332 patients at follow-up [18]. In a study by Tuomainen et al., the mean improvements in the same measures for 70 patients at follow-up were 14.9, 3.1 and 2.3 points, respectively [19]. Karlsson et al. reported a mean improvement in ODI of 14 points in 49 patients at 5 years post-surgery [20].

The 13.9% rate for mild + CMM patients undergoing any SLSI procedure (surgical decompression, fusion, interspinous spacer implantation, neurostimulation, adhesiolysis, additional mild Procedure) by their 3-year follow-up increased to 18.1% at 5 years post-treatment. When considering only surgical treatments of decompression and fusion, the intervention rate for this group at 5 years is 6.9%. By comparison, in a retrospective review of patient records, Mekhail et al. reported a 12% rate of subsequent surgical decompression in 75 patients by 5 years after receiving the mild Procedure [21]. The rates of subsequent surgical interventions (decompression and/or fusion) reported by Hermansen et al., Tuomainen et al. and Karlsson et al. at 5 years following open surgical decompression were 9.7%, 15.7% and 19%, respectively [[18], [19], [20]].

There were no significant differences in 5-year outcome improvements between the group of patients who were ≥65 years of age and those who were younger at the time of treatment. These results augment the findings of Mekhail et al. comparing outcomes of the mild Procedure in patients ≥65 years to those <65 years at 1-year follow-up, finding no statistically significant differences between the two groups for all measures [22]. The results of this subset analysis also reflect the outcomes found by Hee and Wong (8-year follow-up) [23] and Athiviraham et al. (2-year follow-up) [24], who concluded that the outcomes of decompression laminectomy, with or without fusion, were not influenced by patient age. The 23.7% rate of any SLSI experienced by the older mild + CMM patients compares favorably to the results reported by Staats et al. in a 2-year Medicare claims study of the mild Procedure compared to the use of interspinous spacers for LSS treatment [11]. Using a similar definition of SLSI procedures as in the MOTION study, the authors found a 24.9% rate of subsequent interventions for patients initially receiving the mild Procedure using real-world data from a population at least 65 years of age. The lack of significant differences found in this subset analysis of the two age groups for both functional outcomes and SLSI, together with the absence of adverse events, illustrates that the mild Procedure in combination with CMM is a safe and durable treatment option for LSS in both younger and older patients (see Table 5).

The improvement of all outcomes for the crossover group seen at 4 years following the mild Procedure was not significantly different from the 4-year values seen in the mild + CMM group (Table 4). These long-term results mirror the findings of Pryzbylkowski et al., who found that improvement in outcomes in the short-term (3 months) were not statistically different between patients receiving the mild Procedure as an early intervention and those who delayed their mild treatment by first undergoing multiple epidural steroid injections [25].

**Table 4:** Outcome improvements for the crossover group between 2-, 3- and 4-year follow-up visits.

Outcome Improvement (Mean ± SD) P-value (within group compared to baseline)	2-Year	3-Year	4-Year
Sample Size:	N = 18	N = 15	N = 18
ODI	17.0 ± 19.6	19.2 ± 16.0	19.2 ± 22.0
p-value	0.0019	<0.0001	<0.0001
NPRS			
Back	3.5 ± 2.9	3.5 ± 2.6	3.2 ± 2.4^‡^
p-value	<0.0001	0.0005	<0.0001
Leg	4.9 ± 2.3	4.7 ± 2.9	4.6 ± 2.3^‡^
p-value	<0.0001	<0.0001	<0.0001
ZCQ			
Symptom Severity	0.9 ± 0.7	0.8 ± 0.5	0.8 ± 0.9
p-value	<0.0001	<0.0001	<0.0001
Physical Function	0.5 ± 0.7	0.6 ± 0.6	0.6 ± 0.7
p-value	0.0111	0.0021	0.0052
‡ One subject did not complete the assessment			
**Sample Size:**	**N=15**^a^	**N=13**^a^	**N=16**^a^
Walking Tolerance Test	220%	240%	170%
p-value	0.0002	0.0017	0.0002

a Not all patients who completed 5-year follow-up questionnaires performed the WTT.

**Table 5:** Outcome Improvement Comparison At 4-Year Follow-up Between mild + CMM and Crossover Patients.

Outcome Improvement (Mean ± SD) P-value (within group)	mild + CMM	Crossover	P-Value Between Groups
Sample Size:	N = 44	N = 18	
ODI	19.4 ± 20.5	19.2 ± 22.0	0.2979
p-value	<0.0001	<0.0001	
NPRS			
Back	2.3 ± 2.9	3.2 ± 2.4^‡^	0.7577
p-value	<0.0001	<0.0001	
Leg	4.7 ± 2.9	4.6 ± 2.3^‡^	0.3613
p-value	<0.0001	<0.0001	
ZCQ			
Symptom Severity	0.8 ± 0.9	0.8 ± 0.9	0.6832
p-value	<0.0001	<0.0001	
Physical Function	0.6 ± 0.7	0.6 ± 0.7	0.2757
p-value	<0.0001	0.0052	
‡ One subject did not complete the assessment			
**Sample Size:**	**N=44**	**N=16**	
Walking Tolerance Test	322%	170%	0.0883
p-value	<0.0001	0.0002	

* Not all patients who completed 5-year follow-up questionnaires performed the WTT.

### 4.1 Limitations

In many instances, the standard deviations of the study results are larger than the associated means, which can result from high data variability [26]. It is recognized that patients with chronic pain can exhibit intraindividual variability in reporting pain and functional outcome measures [27,28] and may be a contributor to the data variability. The use of several outcome measures helps to provide a comprehensive assessment of the treatment effect in this patient population, however [29], with the universal improvement of all outcome measures strongly supporting the clinical importance of the mild Procedure. An ad hoc analysis using minimal clinically important difference (MCID) was performed to verify that the treatment effect of the mild procedure was meaningful even in the presence of these large standard deviations. While the assessment of MCID was not included in the study protocol, preventing the calculation of anchor-based results, a 10-point improvement in ODI has previously been used in assessing results of low back pain [30,31]. The percentage of patients meeting this ODI MCID in the MOTION study at 5 years is 69.2% These results compare favorably with the 72.4% meeting the MCID reported for the MiDAS ENCORE study of the mild treatment at 2-year follow-up (n = 99).

While long-term prospective longitudinal studies have the benefits of a consistent patient cohort and minimized recall bias, they are prone to patient attrition often due to disallowed treatments during the follow-up period and general loss to follow-up [32]. For the mild + CMM group, the 39 mITT patients participating at the 5-year follow-up represents a 22% subject loss over 3 years based on the 50 patients initially agreeing to extended follow-up, which serves as the primary limitation of this extended follow-up study. The degree of subject loss may have been influenced by the study protocol change to extend the original 2-year follow-up endpoint to 5 years, as the patients in this study were expecting the follow-up visits to last only 2 years when enrolling. A comparable subject loss rate of 25.7% between 2- and 5-year follow-up periods was seen in a study of the Superion® interspinous spacer for treatment of LSS [[33], [34], [35]]. Designed to last just two years to support a U.S. FDA regulatory submission, extended follow-up for the Superion study was mandated by the FDA as part of the Superion device approval [35,36]. Another limitation of this extended follow-up of the MOTION study is a lack of control of the types of allowed post-procedure CMM therapies; while reflecting the real-world environment of this clinical investigation, allowing a wide range of therapies could induce unrecognized bias into the results.

## 5 Conclusion

The improvements in outcomes seen for mild + CMM patients at 5 years post-treatment provide evidence of the long-term durability of the mild Procedure as a safe and effective treatment for LSS with neurogenic claudication secondary to hypertrophic ligamentum. The minimal increase in SLSIs in this patient group since the 3-year follow-up further demonstrates the ability of this procedure to provide lasting symptom relief, with the low rate of subsequent surgical interventions comparing favorably to those seen for index surgical decompression procedures. Analysis of outcomes by age augment previously reported results showing that the mild Procedure is effective both in younger patients and in those who are 65 years of age or older. Further, a separate subset analysis adds long-term evidence for effective LSS symptom relief with the mild Procedure even after delaying treatment up to a year by initial pursuit of conventional medical management.

## Conflicts of interest

Timothy Deer is the research principal investigator on this study and is a consultant for Abbott, Saluda, Transloc, Vivex, PainTEQ, Saluda, and Spinal Simplicity. He has research funding from Abbott, Vivex, and Saluda. Mayank Gupta is a consultant for Curonix and Vertex Pharmaceuticals. He has research funding from Nevro Corp, Averitas Pharma, Stryker, Boston Scientific, Stratus Medical, Saluda Medical, and Spinal Simplicity. Sherif Costandi has received research funding from Stryker (Paid to the Cleveland Clinic). Timothy Chafin has received research funding from Stryker (Paid to ECU Health Roanoke-Chowan Hospital). Sayed Wahezi is a consultant for Boston Scientific and key opinion leader for TEXNEX. He has research funding from Stryker (Paid to Biomedical Research Alliance of New York). Samyadev Datta has received research funding from Stryker (Paid to Holy Name Medical Center). Dawood Sayed is a consultant for Abbott, Medtronic, Nevro, SPR, and Painteq. He has research funding from Stryker (Paid to University of Kansas Medical Center).

## Appendix

The investigators who enrolled patients in this study are listed below by their affiliated institution, with the institutions presented in order from the highest to the lowest number of enrolled patients. Cleveland Clinic Foundation – Cleveland, OH: S. Costandi, J. Xu; Michigan Pain Specialists, Ann Arbor, MI: E. Washabaugh, J. Chatas; Roanoke-Chowan Pain Management, Ahoskie, NC: B. Chafin; University Hospitals Cleveland Medical Center – Cleveland, OH: A. Kabbara; Montefiore Rehab Center, Bronx, NY: S. Wahezi; Pennsylvania Pain & Spine Institute – Chalfont, PA: H. Qu; The Spine and Nerve Center of The Virginias, Charleston, WV: C. Kim; SIMED Health – Gainesville, FL: Q. Nguyen; Michigan Interventional Pain Center, Brownstown Township, MI: R. Haladjian; Holy Name Medical Center – Teaneck, NJ: S. Datta; Menorah Medical Center – Overland Park, KS: D. Kloster; Kansas Pain Management/Neuroscience Research Center – Overland Park, KS: M. Gupta; Spine & Pain Institute – Lakeland, FL: N. Jassal; Newport Beach Headache and Pain, Newport Beach, CA: R. Paicius; University of Kansas Medical Center Research Institute – Kansas City, KS: D. Sayed; The Pain Medicine & Rehabilitation Center – Seymour, IN: A. Alexander; Centura Health – Colorado Springs, CO: W. Lippert; Central Arkansas Veterans Healthcare System – Little Rock, AR: M. Yousaf; Precision Spine Center – Tyler, TX: D. Griffith, A. Calodney.

## References

[1] Sakai Y., Wakao N., Matsui H., Osada N., Watanabe T., Watanabe K. (2024 Apr 3). Insulin resistance as a risk factor for Flavum Hypertrophy in lumbar spinal stenosis. Spine Surg Relat Res.

[2] Ravindra V.M., Senglaub S.S., Rattani A., Dewan M.C., Härtl R., Bisson E., Park K.B., Shrime M.G. (2018 Dec). Degenerative lumbar spine disease: estimating global incidence and worldwide volume. Glob Spine J.

[3] Lurie J., Tomkins-Lane C. (2016 Jan 4). Management of lumbar spinal stenosis. BMJ.

[4] Deer T.R., Grider J.S., Pope J.E., Lamer T.J., Wahezi S.E., Hagedorn J.M., Falowski S., Tolba R., Shah J.M., Strand N., Escobar A., Malinowski M., Bux A., Jassal N., Hah J., Weisbein J., Tomycz N.D., Jameson J., Petersen E.A., Sayed D. (2022 May 5). Best practices for minimally invasive lumbar spinal stenosis treatment 2.0 (MIST): consensus guidance from the American Society of pain and neuroscience (ASPN). J Pain Res.

[5] Chopko B., Caraway D.L. (2010 Jul-Aug). MiDAS I (mild Decompression Alternative to open Surgery): a preliminary report of a prospective, multi-center clinical study. Pain Physician.

[6] Antony A., Stevenson J., Trimble T., Block J. (2022 Dec). Perspective: a proposed diagnostic and treatment Algorithm for management of lumbar spinal stenosis: an integrated team approach. Pain Physician.

[7] Soto E., Esposito M. (2024). Treatment of lumbar spinal stenosis with neurogenic claudication: an algorithmic approach for the pain physician. J Clin Anesthesia Res.

[8] Zhang X.Y., Zhao J.L., Wang Y.J., Luan J., Lin H.Q., Wang D. (2025 Mar). The efficacy of the Minimally Invasive Lumbar Decompression (MILD®) procedure: a PRISMA-compliant systemic review and meta-analysis. Pain Physician.

[9] Orhurhu V., Brancolini S., Zheng D., Snyder S., Jevotovsky D.S., Chopra H., Sahni S., Li N., D'Souza R.S., Evankovich M., Lynch B., Farrell M.E., Alter B.J., Emerick T. (2025 Jul 11). Minimally Invasive Lumbar Decompression (MILD) in patients with lumbar spinal stenosis: a systematic review of randomized and prospective trials. J Pain Res.

[10] Schomer D.F., Solsberg D., Wong W., Chopko B.W. (2011 Aug 31). Mild(®) lumbar decompression for the treatment of lumbar spinal stenosis. NeuroRadiol J.

[11] Staats P.S., Hagedorn J.M., Reece D.E., Strand N.H., Poree L. (2023 Sep). Percutaneous image-guided lumbar decompression and interspinous spacers for the treatment of lumbar spinal stenosis: a 2-year Medicare Claims Benchmark Study. Pain Pract.

[12] Staats P.S., Chafin T.B., Golovac S., Kim C.K., Li S., Richardson W.B., Vallejo R., Wahezi S.E., Washabaugh E.P., Benyamin R.M., MiDAS ENCORE Investigators (2018 Oct). Long-Term safety and efficacy of minimally invasive lumbar decompression procedure for the treatment of lumbar spinal stenosis with neurogenic claudication: 2-Year results of MiDAS ENCORE. Reg Anesth Pain Med.

[13] Deer T., Kim C., Wahezi S.E., Qu H., Sayed D. (2021 Jun 10). MOTION Study investigators. Objective real-world outcomes of patients suffering from painful neurogenic claudication treated with the *mild®* procedure: interim 6-Month report of a randomized controlled trial. J Pain Res.

[14] Deer T.R., Costandi S.J., Washabaugh E., Chafin T.B., Wahezi S.E., Jassal N., Sayed D. (2022 Apr 8). The MOTION Study: a randomized controlled trial with objective real-world outcomes for lumbar spinal stenosis patients treated with the mild® procedure: One-Year results. Pain Med.

[15] Deer T.R., Chafin T.B., Costandi S.J., Qu H., Kim C., Jassal N., Patel K., Calodney A. (2024 Jan). The MOTION study: Two-year results of a real-world randomized controlled trial of the mild® procedure for treatment of lumbar spinal stenosis. Pain Pract.

[16] Costandi S.J., Deer T.R., Chafin T.B., Kim C. (2025 Mar 12). Three-year results of the MOTION randomized controlled trial for treatment of lumbar spinal stenosis using the percutaneous mild® procedure. Interv Pain Med.

[17] Jain S., Deer T., Sayed D., Chopra P., Wahezi S., Jassal N., Weisbein J., Jameson J., Malinowski M., Golovac S. (2020 Sep). Minimally invasive lumbar decompression: a review of indications, techniques, efficacy and safety. Pain Manag.

[18] Hermansen E., Indrekvam K., Franssen E., Myklebust T.Å., Austevoll I.M., Hellum C., Storheim K., Bånerud I.F., Ebbs E.K., Aaen J., Banitalebi H., Brox J.I., Weber C., Solberg T., Hjulstad A., Brisby H. (2025 May). ISSLS Prize in Clinical Science 2025: a randomized trial on three different minimally invasive decompression techniques for lumbar spinal stenosis. Five years follow-up from the NORDSTEN-SST. Eur Spine J.

[19] Tuomainen I., Aalto T., Pesonen J., Rade M., Pakarinen M., Leinonen V., Kröger H., Airaksinen O. (2020 Sep). Unfolding the outcomes of surgical treatment of lumbar spinal stenosis-a prospective 5- and 10-year follow-up study. Eur Spine J.

[20] Karlsson T., Försth P., Öhagen P., Michaëlsson K., Sandén B. (2024 Jul 1). Decompression alone or decompression with fusion for lumbar spinal stenosis: five-year clinical results from a randomized clinical trial. Bone Jt J.

[21] Mekhail N., Costandi S., Nageeb G., Ekladios C., Saied O. (2021 Nov). The durability of minimally invasive lumbar decompression procedure in patients with symptomatic lumbar spinal stenosis: Long-term follow-up. Pain Pract.

[22] Mekhail N.A., Costandi S.J., Armanyous S., Vallejo R., Poree L.R., Brown L.L., Golovac S., Deer T.R. (2020 Jun 4). The impact of age on the outcomes of minimally invasive lumbar decompression for lumbar spinal stenosis. Med Devices (Auckl).

[23] Hee H.T., Wong H.K. (2003 Apr). The long-term results of surgical treatment for spinal stenosis in the elderly. Singap Med J.

[24] Athiviraham A., Wali Z.A., Yen D. (2011 Jul). Predictive factors influencing clinical outcome with operative management of lumbar spinal stenosis. Spine J.

[25] Pryzbylkowski P., Bux A., Chandwani K., Khemlani V., Puri S., Rosenberg J., Sukumaran H. (2022 Mar). Minimally invasive direct decompression for lumbar spinal stenosis: impact of multiple prior epidural steroid injections. Pain Manag.

[26] Barde M.P., Barde P.J. (2012 Jul). What to use to express the variability of data: standard deviation or standard error of mean?. Perspect Clin Res.

[27] Whitaker M.M., Odell D., Deboeck P.R., Stefanucci J.K., Okifuji A. (2024 Aug). Increased pain variability in patients with chronic pain: a role for pain catastrophizing. J Pain.

[28] Rogers A.H., Smit T., Bakhshaie J., Zvolensky M.J. (2025 Dec). Impact of intraindividual pain variability on functional pain outcomes among adults with chronic pain: an ecological momentary assessment study. J Behav Med.

[29] Worzer W., Theodore B., Rogerson M. (2008). Interpreting the clinical significance of pain questionnaires. Pract Pain Manag.

[30] Hägg O., Fritzell P., Nordwall A., Swedish Lumbar Spine Study Group (2003 Feb). The clinical importance of changes in outcome scores after treatment for chronic low back pain. Eur Spine J.

[31] Ostelo R.W., Deyo R.A., Stratford P., Waddell G., Croft P., Von Korff M., Bouter L.M., de Vet H.C. (2008 Jan 1). Interpreting change scores for pain and functional status in low back pain: towards international consensus regarding minimal important change. Spine (Phila Pa 1976).

[32] Caruana E.J., Roman M., Hernández-Sánchez J., Solli P. (2015 Nov). Longitudinal studies. J Thorac Dis.

[33] Patel V.V., Whang P.G., Haley T.R., Bradley W.D., Nunley P.D., Miller L.E., Block J.E., Geisler F.H. (2014 Jul 5). Two-year clinical outcomes of a multicenter randomized controlled trial comparing two interspinous spacers for treatment of moderate lumbar spinal stenosis. BMC Muscoskelet Disord.

[34] Nunley P.D., Patel V.V., Orndorff D.G., Lavelle W.F., Block J.E., Geisler F.H. (2017 Sep 6). Five-year durability of stand-alone interspinous process decompression for lumbar spinal stenosis. Clin Interv Aging.

[35] Investigating Superion™ In Spinal Stenosis ClinicalTrials.gov identifier: NCT00692276. NCT00692276.

[36] Miller L.E., Block J.E. (2012). Interspinous spacer implant in patients with lumbar spinal stenosis: preliminary results of a multicenter, randomized, controlled trial. Pain Res Treat.

